# Differential Microfluidic Sensors Based on Dumbbell-Shaped Defect Ground Structures in Microstrip Technology: Analysis, Optimization, and Applications

**DOI:** 10.3390/s19143189

**Published:** 2019-07-19

**Authors:** Paris Vélez, Jonathan Muñoz-Enano, Marta Gil, Javier Mata-Contreras, Ferran Martín

**Affiliations:** 1 CIMITEC, Departament d’Enginyeria Electrònica, Universitat Autònoma de Barcelona, 08193 Bellaterra, Spain; 2 Departamento de Ingenieria Audiovisual y Comunicaciones, Universidad Politécnica de Madrid, 28031 Madrid, Spain; 3 Departamento de Ingeniería de Comunicaciones, Universidad de Málaga, 29016 Málaga, Spain

**Keywords:** microwave sensors, differential sensors, fluidic sensors, dielectric characterization, microstrip technology, defect ground structure (DGS)

## Abstract

A microstrip defect ground structure (DGS) based on a pair of dumbbell-shaped slots is used for sensing. The device is a differential sensor consisting of a pair of mirrored lines loaded with a dumbbell-shaped DGS, and the output variable is the cross-mode transmission coefficient. Such a variable is very sensitive to asymmetries in the line pair, e.g., caused by an asymmetric dielectric load in the dumbbell-shaped DGSs. Therefore, the sensor is of special interest for the dielectric characterization of solids and liquids, or for the measurement of variables related to complex permittivity changes. It is shown in this work that by adding fluidic channels on top of the dumbbell-shaped DGSs, the device is useful for liquid characterization, particularly for the measurement of solute concentration in very diluted solutions. A sensitivity analysis useful for sensor design is carried out in this paper.

## 1. Introduction

Defect ground structures (DGSs) are slot patterns of different shapes that have been exhaustively used in microwave engineering in applications as diverse as filters, antennas, and sensors [1,2,3,4,5,6]. In this work, the focus is on dumbbell-shaped DGSs [1] and their application to the design of novel differential sensors sensitive to changes in the complex dielectric constant of the so-called sample under test (typically a solid slab or a liquid). The proposed sensor consists of a pair of mirrored microstrip lines, each one loaded with a dumbbell-shaped DGS transversally oriented to the axis of the lines. The dumbbell-shaped DGS behaves as a series-connected parallel resonant tank, providing a notch in the transmission coefficient of each line, with the position and depth being dependent on the complex permittivity of the region surrounding the resonator. However, in the proposed sensor, the working principle is symmetry disruption [7,8,9,10,11,12,13,14,15,16,17,18,19,20,21,22,23,24,25,26], rather than frequency variation (the most usual working principle in resonator-based sensors [27,28,29,30,31,32,33,34,35]). 

The advantage of using symmetry disruption for sensing is the major robustness against cross sensitivities caused by environmental changes (e.g., temperature or moisture) [8]. Most sensors based on symmetry properties utilize coupling modulation [7,8,9,10,11,12,13,14,15,16] or frequency splitting [15,16,17,18,19,20,21,22]. Recently, it has been demonstrated that the cross-mode transmission coefficient is very useful for detecting tiny alterations of symmetry in a pair of lines [24,25], and differential sensors based on several types of resonant elements have been reported [24,25,26]. In [24,25,26,36,37], the resonant elements are metamaterial-inspired resonators coupled to the pair of lines, and fluidic channels are located on top of the most sensitive region of such resonant elements, in order to detect changes in the dielectric properties of liquids (liquid under test, LUT), as compared to a reference (REF) liquid. The method has been demonstrated to be useful for the measurement of the complex dielectric constant of liquids [25], as well as for the determination of solute content in extremely diluted solutions. For instance, in [37], the measurement of NaCl concentrations in deionized (DI) water with a resolution as small as 0.125 g/L and sensitivity of 0.034 (g/L)^−1^ was reported in a complementary split ring resonator (CSRR)-based sensor. It is also worth mentioning the application of the split ring resonator (SRR)-based differential sensor of [26], used for the measurement of the total electrolyte concentration in urine samples.

Despite the fact that a good sensor performance was achieved in [26,37], a sensitivity analysis, providing design guidelines for the resonant elements, is not straightforward when considering either SRRs or CSRRs. By contrast, in [25,36], where the open complementary split ring resonators (OCSRRs) and open split ring resonators (OSRRs) were the considered resonant elements, such analysis was achieved, and important hints for sensor design were obtained. For example, it was concluded in [36] that for sensitivity optimization, high inductance and small capacitance of the resonant element (OSRR) were required.

In the present paper, we focus on a differential sensor where the output variable is also the cross-mode transmission coefficient, but based on a pair of lines loaded with dumbbell-shaped DGSs. Such DGS can be modeled by a series-connected parallel resonator, and a simple sensitivity analysis can be carried out, as it will be shown. On the basis of this analysis, the dumbbell-shaped DGS has been designed for sensitivity optimization. Then, by adding fluidic channels, the structure has been applied to the measurement of the electrolyte concentration in very diluted solutions, and to the determination of the complex dielectric constant in mixtures of deionized (DI) water and isopropanol.

## 2. The Proposed Sensor, Functionality, Circuit Model, and Analysis

**Figure 1:**
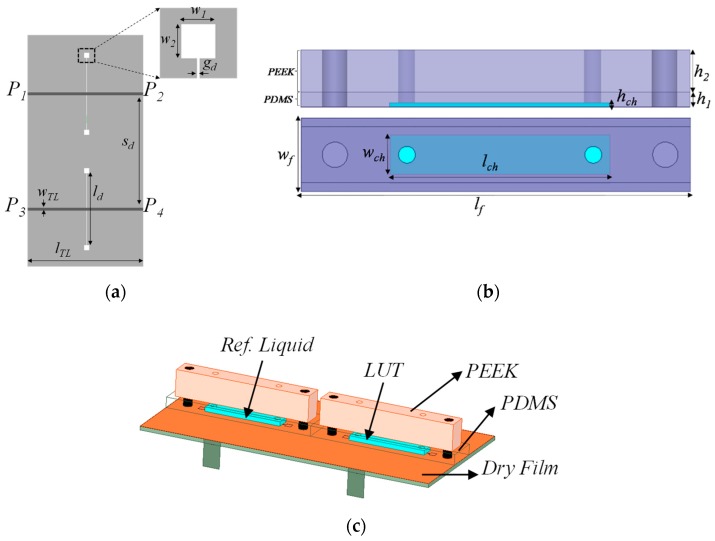
The proposed dumbbell defect ground structure (DGS)-based sensor. (**a**) Topology of the microwave part; (**b**) lateral and top views of the mechanical and fluidic parts; (**c**) complete three-dimensional view. Relevant dimensions are (in mm): *w*_1_ = *w*_2_ = 2, *w_TL_* = 1.14, *l_LT_* = 50, *l_d_* = 28, *g_d_* = 0.2, and *S_d_* = 44. Channel dimensions are (in mm): *h_ch_* = 1.5, *l_ch_* = 26, *w_ch_* = 4.6, *l_f_* = 46, *w_f_* = 12.6, *h*_1_ = 3, and *h*_2_ = 9. The considered substrate is Rogers RO3010 with a dielectric constant *ε_r_* = 10.2, thickness *h* = 1.27 mm, and loss tangent tan*δ* = 0.0035. In (a), the ground plane is depicted in light grey.

The sensor consists of two parts: the microwave structure and the fluidic/mechanical part (Figure 1). The former is a pair of microstrip lines, each one loaded with a dumbbell DGS (etched in the ground plane). The fluidic/mechanical part is composed of the fluidic channels (fabricated by means of Polydimethylsiloxane-PDMS-polymer), plus the accessories required to provide mechanical stability (Polyether Ether Ketone-PEEK-material) and to inject the liquids into the channels. The fluidic channels are placed in contact with the ground plane, specifically on top of the capacitive regions of the dumbbell DGSs, with the most sensitive parts to the effects of liquids being in contact with them. Similar to the sensors reported in [25,26,36,37], the proposed sensor is based on symmetry disruption, and the output variable is the cross-mode transmission coefficient. Due to substrate absorption, a dry film of a clear polyester, with an estimated thickness of 50 μm and dielectric constant of 3.5, has been deposited on top of the dumbbell DGS, which is the sensing region of the sensor (this somehow degrades the sensitivity, but the presence of such a film is necessary to avoid absorption by the substrate). The sensor is able to detect differences between a reference liquid, injected into one of the channels (REF channel), and the liquid under test (LUT), injected into the other channel (LUT channel), manifested as a non-negligible cross-mode transmission coefficient. Note that if symmetry is preserved (identical liquids in both channels), mode conversion is not possible, and thereby the cross-mode transmission coefficient is ideally null.

**Figure 2:**
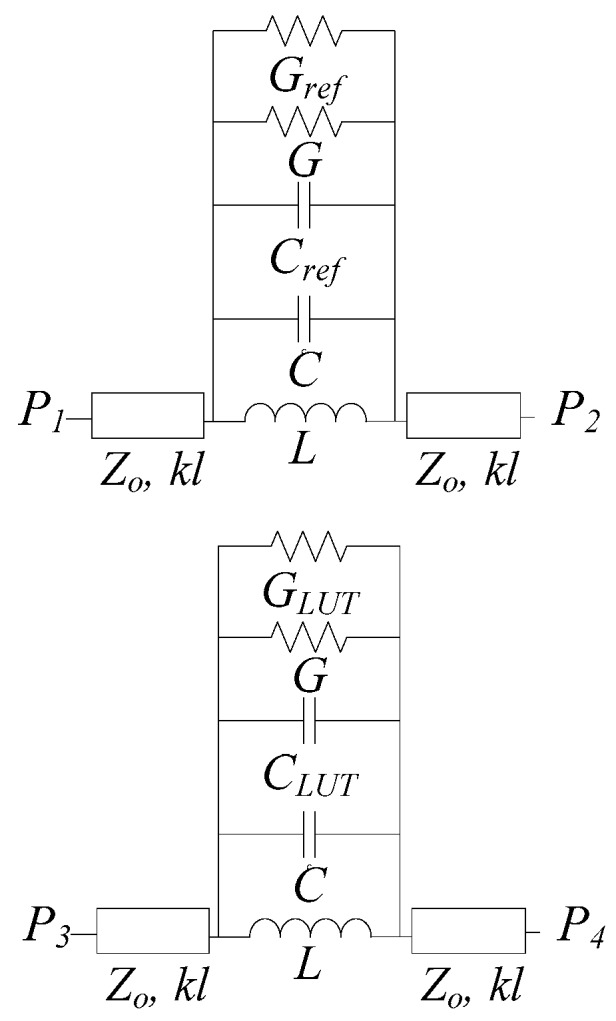
Equivalent circuit model of the proposed sensor.

The equivalent circuit model of the sensor is depicted in Figure 2. The dumbbell DGSs are modeled as series-connected parallel resonant tanks. The reactive elements *L* and *C* are the inductance and capacitance, respectively, of the dumbbell DGSs without liquid in the channels, whereas *G* accounts for the conductance, mainly related to substrate losses (provided that such conductance does not take into account the effects of the liquid in the channel). By introducing liquid into the channels, the elements that are expected to experience variation are the capacitance and conductance of the dumbbell DGSs. Therefore, we have included *C*_ref_ and *G*_ref_ to account for the effects of the liquid in the REF channel (if it is present), whereas *C*_LUT_ and *G*_LUT_ are the corresponding element values for the LUT channel. Finally, the transmission lines are described by their characteristic impedance, *Z*_0_, and electrical length *kl*, with *l* being the physical length and *k* the phase constant.

**Figure 3:**
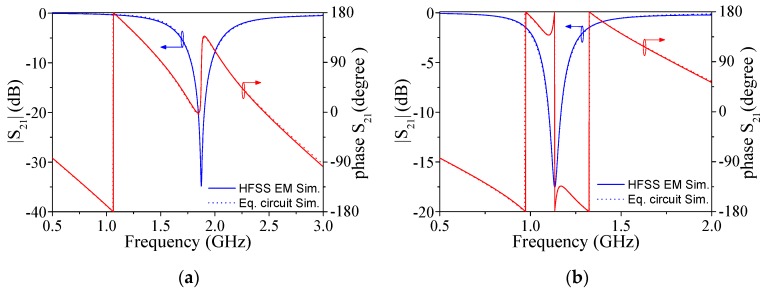
Transmission coefficient (magnitude and phase) of the reference (REF) channel without liquid (a), and with deionized (DI) water (b), inferred from electromagnetic and circuit simulation. The element values of the equivalent circuit model are (in reference to Figure 2): *Z*_0_ = 50 Ω, *kl* = 78.51°, *L* = 2.40 nH, *C* = 2.51 pF, *C*_ref_ = 5.74 pF, *G =* 0.18 mS, and *G*_ref_ = 1.31 mS. Note that in the circuit simulations of (a), *C*_ref_ = 0 pF and *G*_ref_ = 0 mS, as corresponds to the absence of any liquid in the channel.

Model validation has been carried out by comparing electromagnetic simulations (inferred by means of ANSYS HFSS) with circuit simulations (obtained by means of the schematic simulator included in Keysight ADS). Since the REF and LUT lines are uncoupled, it suffices to consider the simulation (circuit and electromagnetic) of any of the lines. The absence of coupling between both lines has been verified from the electromagnetic simulation of the single-ended S-parameters, which are not shown (where it has been found that the S-parameters involving ports of both lines are negligible). Particularly, we have considered the REF line with an empty channel (i.e., with air). The frequency response (transmission coefficient) inferred from *HFSS* simulation is depicted in Figure 3, where it is compared with the circuit simulation. The extracted parameters are indicated in the caption of Figure 3 (note that the presence of the channel and dry film has been taken into account in the electromagnetic simulations from which parameters have been extracted). Excellent agreement in both the magnitude and phase responses can be appreciated, indicative of the validity of the model. We have also simulated the REF line by considering the channel full of DI water. For that purpose, the complex dielectric constant of water has been introduced in the *HFSS* simulator. The response is also depicted in Figure 3. The extracted parameters with an unloaded channel, i.e., *C*, *L*, and *G*, have been maintained, whereas, to include the effects of DI water in the channel, *C*_ref_ and *G*_ref_ have been adjusted (the values are also indicated in the caption of Figure 3). The circuit response corresponding to these circuit parameters is also included in Figure 3, and again, the agreement with the full wave electromagnetic simulation is very good. The presence of DI water in the channel has the effect of decreasing the resonance frequency of the dumbbell DGS and the notch magnitude. This is an expected result as far as the high dielectric constant of DI water significantly increases the overall capacitance of the dumbbell DGS, and the high dissipation factor of DI water (corresponding to a high value of the imaginary part of the complex dielectric constant, or loss tangent) enhances the losses of the resonant element.

The cross-mode transmission coefficient of the structure depends on the length and characteristic impedance of the access lines (with impedance *Z*_0_ and electrical length *kl*). However, if the line impedance is set to the reference impedance of the ports, the magnitude of the cross-mode transmission coefficient only depends on the impedance of the dumbbell DGSs, according to [38]:

(1) $$\left|S_{21}^{dc}\right|=\frac{1}{2}\left|\left(S_{21}-S_{43}\right)\right|$$

where *S*_21_ and *S*_43_ are the transmission coefficients of the individual DGS-loaded lines, by excluding the access lines.

(2a) $$S_{21}=\frac{1}{1+\frac{1}{2Z_{0}}Z_{\mathrm{REF}}}$$

(2b) $$S_{43}=\frac{1}{1+\frac{1}{2Z_{0}}Z_{\mathrm{LUT}}}$$

Additionally, the impedance of the dumbbell DGSs, *Z*_REF_ and *Z*_LUT_, for the REF and LUT channels, respectively, are given by

(3a) $$Z_{\mathrm{REF}}=\frac{j\omega L}{j\omega L{G^{\prime }}_{\mathrm{ref}}-\frac{\omega^{2}}{\omega_{\mathrm{ref}}^{2}}+1}$$

(3b) $$Z_{\mathrm{LUT}}=\frac{j\omega L}{j\omega L{G^{\prime }}_{\mathrm{LUT}}-\frac{\omega^{2}}{\omega_{\mathrm{LUT}}^{2}}+1}$$

In Equation (3a) and (3b), we have defined the following variables in order to simplify the notation:

(4a) $${C^{\prime }}_{\mathrm{ref}}=C+C_{\mathrm{ref}}$$

(4b) $${G^{\prime }}_{\mathrm{ref}}=G+G_{\mathrm{ref}}$$

(4c) $${C^{\prime }}_{\mathrm{LUT}}=C+C_{\mathrm{LUT}}$$

(4d) $${G^{\prime }}_{\mathrm{LUT}}=G+G_{\mathrm{LUT}}$$

(4e) $$\omega_{\mathrm{ref}}^{-2}=L{C^{\prime }}_{\mathrm{ref}}$$

(4f) $$\omega_{\mathrm{LUT}}^{-2}=L{C^{\prime }}_{\mathrm{LUT}}$$

Also note that Equation (1) is correct as far as the REF and LUT lines are uncoupled.

Let us consider that the output variable is the cross-mode transmission coefficient evaluated at the resonance frequency of the reference channel, *ω*_ref_. For small perturbations and low losses, *ω*_ref_ and *ω*_LUT_ are not very different, and both *G′*_ref_ and *G′*_LUT_ are small compared to *Y*_0_ ≡ 1/*Z*_0_. Under these circumstances, we can express *S*_21_ and *S*_43_ as

(5a) $${S_{21}|}_{\omega_{\mathrm{ref}}}=\frac{1}{1+\frac{1}{2Z_{0}{G^{\prime }}_{ref}}}≅2Z_{0}{G^{\prime }}_{ref}$$

(5b) $${S_{43}|}_{\omega_{\mathrm{ref}}}=\frac{1}{1+\frac{1}{2Z_{0}{G^{\prime }}_{LUT}-j\frac{\omega_{ref}}{L}\left(\frac{1}{\omega_{ref}^{2}}-\frac{1}{\omega_{LUT}^{2}}\right)}}≅2Z_{0}{G^{\prime }}_{LUT}-j\frac{\omega_{\mathrm{ref}}}{L}\left(\frac{1}{\omega_{ref}^{2}}-\frac{1}{\omega_{LUT}^{2}}\right)$$

and the modulus of the cross-mode transmission coefficient is found to be

(6) $${\left|S_{21}^{dc}\right|}_{\omega_{\mathrm{ref}}}≅Z_{0}\left|G_{\mathrm{ref}}-G_{\mathrm{LUT}}+j\omega_{\mathrm{ref}}\left(C_{ref}-C_{LUT}\right)\right|$$

**Figure 4:**
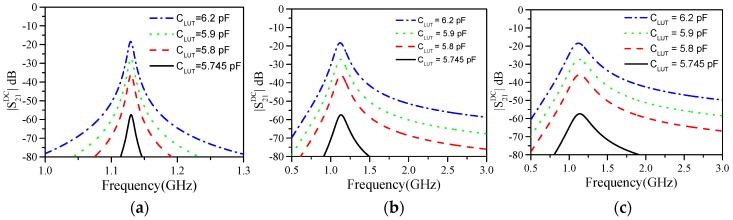
Cross-mode transmission coefficient inferred by circuit simulation for different cases, and the indicated values of *C*_LUT_. (a) *L* = 0.1 nH, *C* = 192.63 pF; (b) *L* = 1.7 nH, *C* = 5.93 pF; (c) *L* = 2.9 nH, *C* = 1.10 pF. In all the cases, *C*_ref_ = 5.74 pF and *G′*_ref_ = *G′*_LUT_ = 1.489 mS.

Since Z_0_ is not a design parameter, the sensitivity of the output variable, |*S*_21_*^DC^*|*_ω_*_ref_, with the differential conductance, *G*_ref_−*G*_LUT_, cannot be controlled. On the other hand, it follows from (6) that the sensitivity of the output variable with the differential capacitance, *C*_ref_−*C*_LUT_, increases with the resonance frequency of the REF channel, *ω*_ref_. It also follows from (6) that if *G*_ref_ = *G*_LUT_, the magnitude of the cross-mode transmission coefficient should not depend on *L* and *C*, provided these elements and *C*_ref_ give a constant value of *ω*_ref_. This is corroborated in Figure 4, where the magnitude of the cross-mode transmission coefficient for different cases is depicted. In all the cases, *ω*_ref_ = 1.13 GHz, but different combinations of *L* and *C* have been considered in order to obtain the above cited value of *ω*_ref_ with the same value of *C*_ref_ = 5.74 pF. Nevertheless, rather than the differential capacitance or conductance, the input variables in a real scenario are, typically, material (liquid in our case) parameters, such as the dielectric constant and the loss tangent, or other variables related to them (e.g., the electrolyte concentration). Therefore, a further step is necessary in order to determine the dependence of the cross-mode transmission coefficient with these material parameters, which will be discussed next.

Let us assume that the capacitive slot of the dumbbell DGS is narrow compared to the thickness of the substrate, and that the substrate is thick enough to consider that the electric field lines generated in the slot region do not reach the air region present at the opposite side of the substrate. Let us also consider that the height of the channels is great enough to guarantee that the electric field lines generated in the dumbbell DGS slot do not extend beyond the region occupied by the liquid present in the channel. Under these conditions, it can be considered that two uniform half-spaces, the substrate and the liquid, surround the slot capacitance. According to [35], *C’*_ref_ and *C’*_LUT_ can be expressed as

(7a) $${C^{\prime }}_{ref}=C\left(\frac{\varepsilon_{r}+\varepsilon_{ref}}{\varepsilon_{r}+1}\right)$$

(7b) $${C^{\prime }}_{LUT}=C\left(\frac{\varepsilon_{r}+\varepsilon_{LUT}}{\varepsilon_{r}+1}\right)$$

Therefore, the differential capacitance that appears in the imaginary part of Equation (6) is

(8) $$C_{ref}-C_{LUT}=\;{C^{\prime }}_{ref}-{C^{\prime }}_{LUT}=\frac{C}{\varepsilon_{r}+1}\left(\varepsilon_{\mathrm{ref}}-\varepsilon_{\mathrm{LUT}}\right)$$

If we now express *C* as the contribution of the substrate and air capacitance, i.e.,

(9) $$C=C_{subs}+C_{air}$$

and we take into account that *C_subs_* = *ε_r_*⋅*C_air_*, it follows that

(10) $$C=C_{subs}\frac{\varepsilon_{r}+1}{\varepsilon_{r}}$$

Therefore, Equation (8) can be expressed as

(11) $$C_{ref}-C_{LUT}=\frac{C_{subs}}{\varepsilon_{r}}\left(\varepsilon_{\mathrm{ref}}-\varepsilon_{\mathrm{LUT}}\right)$$

Since *C_subs_* is proportional to the dielectric constant of the substrate, *ε_r_*, it follows that the sensitivity of the output variable with the differential dielectric constant, *ε*_ref_−*ε*_LUT_, is proportional to *ω*_ref_, and determined by the geometry of the slot, particularly by its length and width. Note that *C_subs_* is roughly proportional to the slot length and it increases by decreasing the slot width. Hence, for sensitivity optimization with regard to the differential permittivity, narrow and long slots for the dumbbell DGS are required.

The relation between the conductance of the REF or LUT liquid and the corresponding loss tangents is given by [35]

(12a) $${\tan\delta }_{ref}=\frac{G_{\mathrm{ref}}}{\left(C_{air}+C_{ref}\right)\omega_{\mathrm{ref}}}=\frac{G_{\mathrm{ref}}(\varepsilon_{r}+1)}{C\varepsilon_{\mathrm{ref}}\omega_{\mathrm{ref}}}$$

(12b) $${\tan\delta }_{LUT}=\frac{G_{\mathrm{LUT}}}{\left(C_{air}+C_{LUT}\right)\omega_{\mathrm{LUT}}}=\frac{G_{\mathrm{LUT}}(\varepsilon_{r}+1)}{C\varepsilon_{\mathrm{LUT}}\omega_{\mathrm{LUT}}}$$

Therefore,

(13) $$G_{ref}-G_{LUT}=\frac{C_{subs}\omega_{\mathrm{ref}}}{\varepsilon_{r}}\left(\varepsilon_{\mathrm{ref}}{\tan\delta }_{\mathrm{ref}}-\varepsilon_{\mathrm{LUT}}{\tan\delta }_{\mathrm{LUT}}\right)$$

where the approximation *ω*_ref_ ≈ *ω*_LUT_ has been used. In this case, it is not possible to express the differential conductance, *G*_ref_ − *G*_LUT_, as proportional to the differential loss tangent. Nevertheless, the inspection of Equation (13) reveals that the differential conductance is proportional to *ω*_ref_ and it is also determined by the slot dimensions (through *C_subs_*), with identical dependence to the differential capacitance.

Introducing Equations (11) and (13) into Equation (6), the cross-mode transmission coefficient is found to be

(14) $${\left|S_{21}^{dc}\right|}_{\omega_{\mathrm{ref}}}≅Z_{0}\frac{C_{subs}\omega_{\mathrm{ref}}}{\varepsilon_{r}}\left|\varepsilon_{\mathrm{ref}}{\tan\delta }_{\mathrm{ref}}-\varepsilon_{\mathrm{LUT}}{\tan\delta }_{\mathrm{LUT}}+j\left(\varepsilon_{ref}-\varepsilon_{LUT}\right)\right|$$

Let us analyze Equation (14) more carefully, or, more precisely, the relevant term providing the sensitivity of the output variable with the differential dielectric constant and loss tangent. Such a term can be expressed as

(15) $$\frac{C_{subs}\omega_{\mathrm{ref}}}{\varepsilon_{r}}=\frac{C_{subs}}{\varepsilon_{r}}{\left[L\left(C_{subs}+C_{air}\right)\frac{\varepsilon_{r}+\varepsilon_{ref}}{\varepsilon_{r}+1}\right]}^{-1/2}$$

and after some straightforward calculation, one obtains

(16) $$\frac{C_{subs}\omega_{\mathrm{ref}}}{\varepsilon_{r}}=\frac{1}{\sqrt{\varepsilon_{r}+\varepsilon_{ref}}}\sqrt{\frac{C_{air}}{L}}$$

That is, for sensitivity optimization, it is convenient to choose a substrate with a small value of the dielectric constant. Nevertheless, if the dielectric constant of the REF liquid, $\varepsilon_{ref}$, is high, (as is usual in liquids), it follows that the dependence of the sensitivity on $\varepsilon_{r}$ is small (since $\varepsilon_{r}$ is obscured by $\varepsilon_{ref})$. On the other hand, sensitivity optimization depends on the ratio *C_air_*/*L*. Increasing the slot length, *l*_d_, has the effect of increasing both *C_air_* and *L*. However, whereas *C_air_* increases roughly proportionally with the length of the slot, *L* does not (it has been corroborated by extracting parameters in several structures where the geometry of the slots has been modified). The result is that the sensitivity increases with the slot length, and for this reason, a dumbbell DGS with a long slot has been considered in the designed sensor. On the other hand, the width of the slot, *g_d_*, does not have any influence on *L*, whereas, by reducing it, *C_air_* increases, thereby enhancing the sensitivity. For this reason, we have set *g_d_* to the minimum value of the available fabrication technology (0.2 mm). 

**Figure 5:**
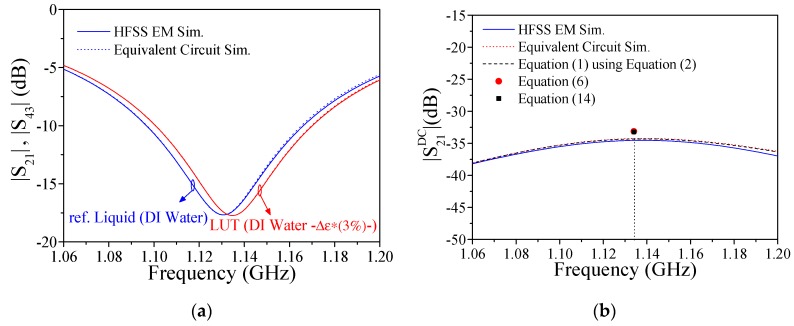
(**a**) Simulated frequency response for the magnitude of the transmission coefficients considering deionized (DI) water in the reference (REF) channel and 3% perturbed DI water in the liquid under test (LUT) channel; (**b**) magnitude of the cross-mode transmission coefficient considering the presented equivalent circuit model, the HFSS electromagnetic (EM) simulations, and Equations (6) and (14).

Equation (14) gives the magnitude of the cross-mode transmission coefficient as a function of the dielectric properties of the REF and LUT liquids (*ε*_ref_, *ε*_LUT_, tan*δ*_ref_, and tan*δ*_LUT_). Considering DI water as the reference liquid (with a complex dielectric constant of 80.66–j4.92 at *ω*_ref_), and the LUT channel full with a hypothetical liquid exhibiting complex dielectric constant variation of 3% (smaller) compared to the one of the REF liquid (i.e., 78.24–j4.77), we have obtained the responses of both lines, and from them, the cross-mode transmission coefficient (Figure 5). The circuit and electromagnetic simulation responses are depicted in the figure. Moreover, Figure 5b includes the results derived from the analytical Equations (6) and (14). Equations (6) and (14) provide an undistinguishable result, which in turn coincides reasonably well with the results inferred from the electromagnetic and circuit simulations at *ω*_ref_. In other words, the analytical (and approximate) formulas predict, in a reasonable way, the value of the cross-mode transmission coefficient at *ω*_ref_. Such good agreement validates the proposed equivalent circuit model (Figure 2), including liquid properties (*C*_ref_, *G*_ref_, *C*_LUT_, and *G*_LUT_), as well as the low-loss and small perturbation assumptions for the considered case. Once the proposed sensor has been presented, analyzed, and optimized, the experimental results will be summarized in the next section. 

## 3. Results

The experimental results obtained by using the proposed sensor for the characterization of electrolyte concentration (particularly NaCl) in DI water and for the measurement of the complex dielectric constant of liquids (mixtures of isopropanol and DI water), are presented in this section.

### 3.1. Electrolyte (NaCl) Concentration Measurements in DI Water

The measurement of electrolyte concentration in urine and/or blood can be used as an indicator of certain pathologies. Electrolytes are cations (e.g., Na^+^, K^+^, and Ca^2+^) and anions (e.g., Cl^−^ and HCO_3_^−^) that result from the dissociation of polar solvents, e.g., NaCl. In this work, the experimental study is focused on the determination of NaCl concentration (the solute) in DI water (the solvent), through the measurement of the maximum value of the cross-mode transmission coefficient. The pure DI water acts as the REF liquid, whereas the LUT is the solution of NaCl in DI water (different levels of NaCl concentration are considered). The proposed sensor is able to detect very small concentrations of NaCl, as it will be shown, thereby providing a very good resolution. 

**Figure 6:**
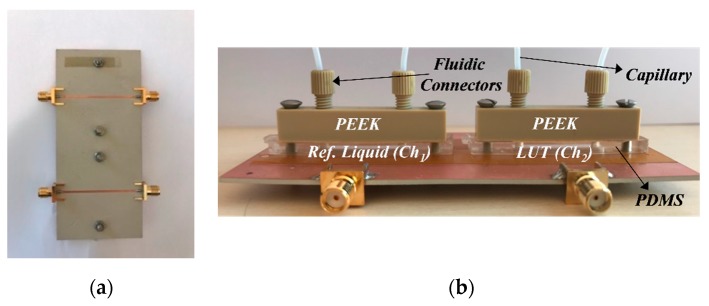
Photographs of the fabricated microwave sensor. The considered materials are indicated. (**a**) Top view (**b**) Lateral view.

**Figure 7:**
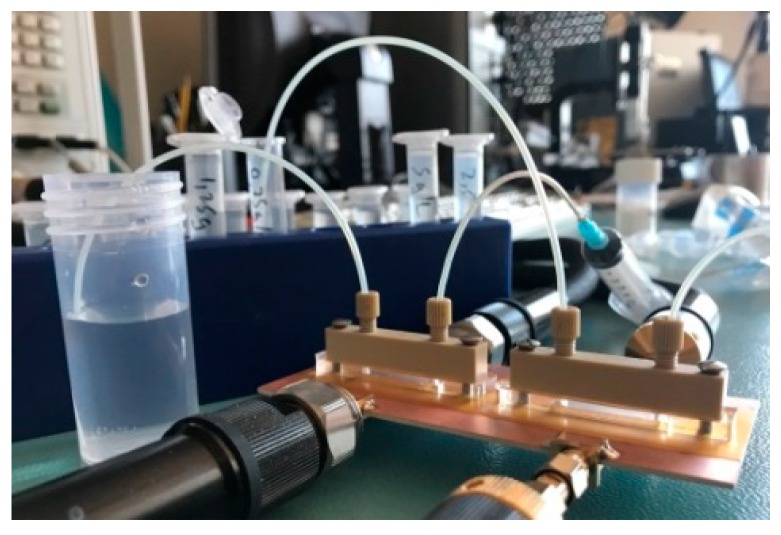
Setup for experimental sensor verification in the laboratory.

The fabricated microwave sensor, including the screws used to assemble the mechanical and fluidic parts, is shown in Figure 6. The considered substrate for the microwave circuitry is the Rogers RO3010 with a dielectric constant *ε_r_* = 10.2, thickness *h* = 1.27 mm, and loss tangent tan *δ* = 0.0035. The different mixtures of DI water and NaCl have been prepared carefully in the laboratory. The measurements have been carried out by the well-known stop-flow technique. This means that the two channels are filled (by syringe) with the REF liquid (DI water) and LUT (DI water with NaCl content), see Figure 7. Then, the flow is stopped for measurement, and the relevant information (maximum cross-mode transmission coefficient, or other potential information of interest, such as the resonance frequency, quality factor, etc.) is recorded and processed (if it is needed). 

**Figure 8:**
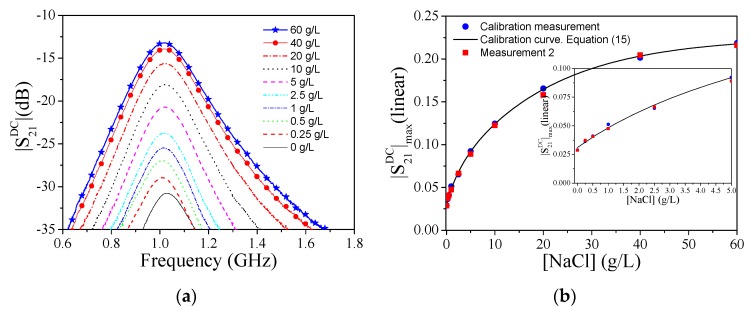
(**a**) Cross-mode insertion loss for different concentrations of NaCl (**b**) Variation of |S_21_^DC^|_max_ with NaCl content.

Following the aforementioned technique, the measured cross-mode transmission coefficient for the different mixtures of DI water and NaCl is plotted in Figure 8a. To evaluate the symmetry of the structure, both channels are filled with the same liquid (the REF liquid). The maximum cross-mode insertion loss (the cross-mode transmission coefficient expressed in dB) for this case is −31.94 dB, which is considered to be a reasonable result, indicating that the structure is quite balanced when identical liquids are present in both channels. It should be mentioned that for proper balance, we first measured the cross-mode transmission coefficient of the structure without fluidic channels, in order to verify that the cross-mode transmission coefficient is very small (ideally null in a perfectly balanced structure). Then, we repeated this procedure after adding the fluidic channels, in order to verify that the structure is also balanced with the presence of the fluidic channels. If necessary, the pressure of the channels on the microwave substrate can be tailored by means of the screws. Once the structure exhibits good balance (typically with a cross-mode insertion loss better than 30 dB), then it is quite robust from a mechanical viewpoint. As the concentration of NaCl in DI water increases, the maximum cross-mode insertion loss also increases, as expected, due to an increasing symmetry imbalance between both channels. The main effect of the presence of electrolytes in DI water is the variation of the dissipation factor (loss tangent or imaginary part of the complex permittivity), due to the fact that electrolytes are ions, significantly contributing to the increase in the conductivity of the solution. The results of Figure 8a, indicating that the frequency of maximum cross-mode insertion loss (for each concentration level) does not experience a significant change, corroborate the previous assertion. It should also be pointed out that concerning environmental parameters, changes in temperature and pressure may alter the response of the lines, but these environmental parameters are seen as a common mode stimulus in a differential sensor like this, and, therefore, their influence is not expected to be important.

The variation of the maximum value of the cross-mode transmission coefficient (|S_21_^DC^|_max_) with the concentration of NaCl (in g/L) is depicted in Figure 8b. The calibration curve (Equation 17) with the correlation coefficient *R*^2^ = 0.99992 is also plotted in the figure. The sensitivity increases as the NaCl concentration decreases (see the zoom inset in Figure 8b). The highest value of the sensitivity has been found to be 0.035 (g/L)^−1^, with a sensor resolution of 0.25 g/L. To evaluate the repetitiveness of the sensor response, a set of mixtures of DI water and NaCl (corresponding to the concentrations of the first campaign) has been injected in aleatory order in the LUT channel. The results are also plotted in Figure 8b, and designated as Measurement 2. The small differences between the calibration curve and the Measurement 2 curve verify the correct functionality and repetitiveness of the sensor.

(17) $$\left[\mathrm{NaCl}\right]\left(\frac{g}{L}\right)=3.425e^{\left(\frac{S_{21}^{DC}}{0.085}\right)}+3.295e^{-4}e^{\left(\frac{S_{21}^{DC}}{0.019}\right)}-4.98$$

### 3.2. Dielectric Characteritzation of Isopropanol in DI Water

The functionality of the proposed microwave sensor also extends to the dielectric characterization of liquids, i.e., the determination of the complex dielectric constant. In this case, the output variable will be the difference between the maximum cross-mode insertion loss ($\Delta {\left|S_{21}^{DC}\right|}_{max}={\left|S_{21}^{DC}\right|}_{max,REF}-{\left|S_{21}^{DC}\right|}_{max,LUT}$) and the difference between the frequencies of the maximum cross-mode insertion loss ($\Delta f_{max}=f_{max,LUT}-f_{max,REF}$) for each measurement. The reason for this is that the complex dielectric constant is composed of real and imaginary parts, thereby requiring two independent output variables for its univocal determination. 

**Figure 9:**
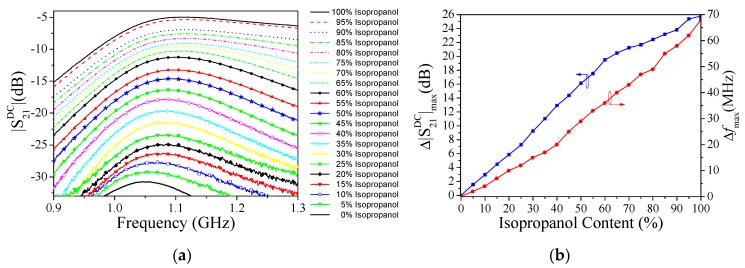
(**a**) Cross-mode insertion loss for different mixtures of isopropanol and deionized (DI) water (**b**) dependence of Δ|*S_21_^DC^*|_max_ and Δ*f*_max_ with isopropanol content.

The dielectric characterization of liquids proceeds as follows. First of all, the maximum cross-mode insertion loss and its frequency position when both channels are loaded with pure DI water (the REF liquid) are recorded. These values correspond to the reference level, namely ${\left|S_{21}^{DC}\right|}_{max,REF}$ and $f_{max,REF}$, respectively. Then, pure isopropanol is injected into the LUT channel and the corresponding values of the maximum cross-mode insertion loss and frequency position are obtained. This is repeated for a mixture of 50% of isopropanol. The cross-mode insertion loss corresponding to these LUTs is depicted in Figure 9a. Knowing the complex dielectric constant (at *ω*_ref_) of pure DI water (80.66–j4.92), of pure isopropanol (13–j5.9), and of a mixture of 50% of isopropanol (32.38−5.38 j), and assuming a linear dependence of the complex dielectric constant with $\Delta {\left|S_{21}^{DC}\right|}_{max}$ and $\;\Delta f_{max}$, it is possible to express the variation of the real and imaginary parts of the complex permittivity with the following linear regression in order to calibrate the sensor:

(18a) $$\Delta \varepsilon^{\prime }=k_{11}\Delta {\left|S_{21}^{DC}\right|}_{max}+k_{12}\Delta f_{max}$$

(18b) $$\Delta \varepsilon^{\prime \prime }=k_{21}\Delta {\left|S_{21}^{DC}\right|}_{max}+k_{22}\Delta f_{max}$$

**Figure 10:**
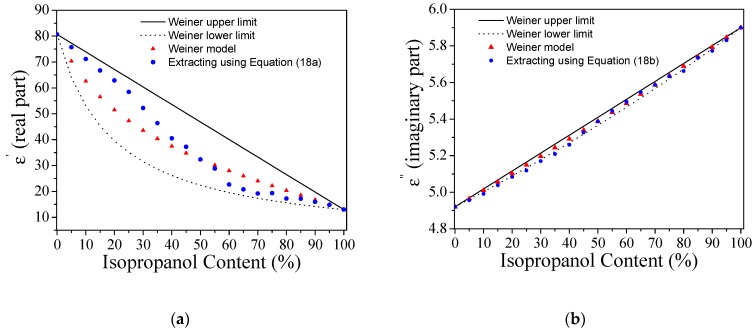
Extracted value for the complex dielectric constant in mixtures of deionized (DI) water/isopropanol. (**a**) Real part. (**b**) Imaginary part. The static Weiner model (upper and lower limits) is also included for comparison purposes.

The unknown variables *k*_11_, *k*_12_, *k*_21_, and *k*_22_ were found to be *k*_11_ = 3.789 dB^−1^, *k*_12_ = 0.441 MHz^−1^, *k*_21_ = 0.009 dB^−1^, and *k*_22_ = 0.010 MHz^−1^. Then, we sequentially injected the different mixtures of DI water and isopropanol into the LUT channel with a volume fraction ranging from 0% to 100% in steps of 5%. All of these measurements are plotted in Figure 9a. The $\;\Delta {\left|S_{21}^{DC}\right|}_{max}$ and $\Delta f_{max}$ for each measurement are shown in Figure 9b. From Equation (18) and the data of Figure 9b, the real and the imaginary part of the complex dielectric constant, as a function of isopropanol content in DI water, have been obtained (Figure 10). In the same figure, the Weiner model [39] has been used to establish the upper and lower limits of the real and imaginary part of the complex dielectric constant for mixtures of both liquids. The calculated values for the complex dielectric constant of mixtures of DI water and isopropanol are between the limits predicted by the Weiner model. These results validate the correct functionality of the proposed sensor for the dielectric characterization of liquids. 

It should also be pointed out that the sensor is useful for monitoring the concentration of isopropanol in DI water, with a resolution of at least 5% of the volume fraction, as derived from the results of Figure 9a. 

## 4. Comparison with Other Microwave Sensor Approaches and Discussion

Let us now compare the proposed sensor with other sensors devoted to measuring the solute content in diluted solutions of DI water. Due to the difficulty in finding other microwave sensors reported in the literature, measuring the same solute (in this case NaCl) and the same, or similar, output variable, we have included comparative works where solutions of glucose (and even heavy metals like Zn) have been considered. As it can be seen in Table 1, the reported solution based on dumbbell DGS resonators offers a good sensor performance (i.e., combination of sensitivity, resolution, and input dynamic range). In references [25,26,40,41], the authors use NaCl as the solute, whereas the other sensors are used for glucose concentration measurement, except [42,43], devoted to measurement of the Zn concentration in DI water. According to Table 1, for the NaCl or glucose concentration, the proposed sensor and the one presented in [26] offer the best sensitivity and resolution, the key sensor parameters. Indeed, the sensitivity of both sensors is comparable, but it should be mentioned that in [26], the considered substrate is *FR4*, with a significantly smaller dielectric constant, compared to the one of the sensor reported in this work. As it has been previously discussed, a small dielectric constant substrate favors sensitivity. Moreover, the measurement of the maximum sensitivity may be somehow inaccurate because it is influenced by the cross-mode insertion loss under balanced conditions (i.e., with the REF liquid in both channels). The achieved value in this work is −31.94 dB, as indicated before, whereas in [26], −38.95 dB was obtained. Although the sensor reported in [26], based on split ring resonators (SRRs), is very competitive, the analysis of SRR-loaded lines for the determination of the sensitivity and the dependence on the main parameters is not straightforward. By contrast, in this paper, we have provided expressions which are useful for sensor design, as far as they predict the output variable as a function of material parameters reasonably well, and provide useful hints for sensitivity optimization (this is indeed the main relevant contribution of the present paper). On the other hand, the sensors reported in [42,43], for Zn concentration measurements, exhibit an extremely good resolution (but a limited dynamic range), at the expense of using functionalized bismuth oxide coatings.

For the measurement of dielectric properties of liquids in the microwave range using resonant elements, the comparison is often based on the relative sensitivity of the resonance frequency with the real part of the complex dielectric constant, defined as

(19) $$S_{av,f}=\frac{1}{f_{0}}\frac{df_{0}}{d\varepsilon_{r}}$$

Note that in the reported differential sensor, the frequency considered as the output variable is *f*_max_, i.e., the frequency where the cross-mode insertion loss is maximized. This frequency does not correspond to the resonance frequency of the dumbbell DGS loaded with the LUT under consideration. Therefore, for a useful comparison, we should consider the relative sensitivity as derived by the variation experienced by the resonance frequency of the LUT channel, *f*_0_, with the relative dielectric constant of the LUT. This relative sensitivity, identified in Table 2 as “single ended (*SE*)”, is excellent, indicating that the dumbbell DGS resonator is very sensitive to changes in the dielectric constant of its surroundings. Nevertheless, we have also included in the table the relative sensitivity derived by considering the variation of *f*_max_ with *ε_r,_*_LUT_, and identified in the table as “Diff.” (which is worse than the previous one). According to these words, if differential sensing is not due, “single-ended measurements” provide a better sensitivity of the resonance frequency with the real part of the dielectric constant of the material under test. The reported sensor provides a good resolution in regard to the volume fraction *(F_v_)* of solute content, regardless of its functionality as a differential or single-ended sensor, and it exhibits the best *SE* sensitivity among all the sensors of Table 2. Moreover, operation in differential mode is convenient due to the major robustness against cross sensitivities, as mentioned before, and it is used in applications of the sensor as a comparator, where real-time monitoring of the differences between a sample under test and a reference sample is typically required.

Note that the analysis of Section 2, providing Equation (14), has been mainly devoted to inferring the design strategy of the dumbbell DGS, in order to enhance sensor sensitivity (i.e., to achieve the maximum variation of the modulus of the cross-mode transmission coefficient at *ω*_ref_ with either the differential dielectric constant or loss tangent). The validity of Equation (14), by considering small perturbations, has been demonstrated in Section 2. However, such an expression has not been used for the determination of NaCl concentration in Section 3a, or for the measurement of the complex dielectric constant in mixtures of isopropanol and DI water in Section 3b. In the former case, the relationship between the loss tangent and the NaCl content in solutions of NaCl in DI water is not easy to infer, so we have opted to obtain the calibration curve depicted in Figure 8b. Moreover, the validity of Equation (14) is constrained to small perturbations. Concerning the determination of the complex dielectric constant in solutions of isopropanol and DI water, not only is the small perturbation approximation not always satisfied, but also, it is not possible to obtain the material parameters (dielectric constant and loss tangent), using Equation (14), from the measurement of the cross-mode transmission coefficient. This is because the determination of two parameters requires two independent measurements. For these main reasons, the interest of Equation (14) is not the analytical determination of the variables of interest, but the deduction of the shape of the dumbbell DGS for sensitivity optimization. Additionally, in the fabricated sensor, a dry film is necessary in order to avoid substrate absorption, and this is an additional limitation concerning the validity of Equation (14). 

Nevertheless, to gain insight into the limitations relative to the validity of Equation (14) in a real scenario (sensor with a dry film and/or subjected to significant imbalances), we have considered the sensor with DI water in the REF channel and with a mixture of isopropanol and DI water in the LUT channel. In such mixtures, we have first considered a volume fraction of 5% isopropanol, corresponding to a relatively small imbalance in the sensor. The material parameters for DI water are *ε*_ref_ = 80.66 and tan*δ*_ref_ = 0.0609; for the considered 5% isopropanol solution, they are *ε*_LUT_ = 77.277 and tan*δ*_LUT_ = 0.0643, as derived from the Weiner model. From the simulation of the transmission coefficient of the individual lines, we have obtained the resonance (notch) frequency for both the REF line, *f*_0,ref_, and LUT line, *f*_0,LUT_. Such frequencies are related to the dielectric constants of the REF and LUT liquids according to

(20) $$\frac{f_{0,\mathrm{ref}}^{2}}{f_{0,\mathrm{LUT}}^{2}}=\frac{\varepsilon_{r}+\varepsilon_{\mathrm{LUT}}}{\varepsilon_{r}+\varepsilon_{\mathrm{ref}}}$$

From the previous expressions, it is possible to derive the dielectric constant of the LUT, provided that *ε*_ref_ is known. The result is *ε*_LUT_ = 77.38, i.e., very close to the nominal value introduced in the simulation (77.277). Then, we have introduced this value, as well as the material parameters of DI water given above, into expression (14). From the simulated value of the cross-mode transmission coefficient at *ω*_ref_, we have isolated the loss tangent of the considered LUT liquid, and the resulting value has been found to be tan*δ*_LUT_ = 0.0630, i.e., in good agreement with the nominal value (0.0643). By repeating the simulation with a dry film with the thickness and dielectric constant indicated in Section 2, the material parameters are found to be *ε*_LUT_ = 78.11 and tan*δ*_LUT_ = 0.0682. That is, the discrepancies are higher, and such discrepancies are even higher when the material parameters are obtained from the measured data (*ε*_LUT_ = 79.63 and tan*δ*_LUT_ = 0.0687). These results indicate that with a 5% volume fraction of isopropanol, the small perturbation approximation is valid, and the substrate, the REF liquid, and the LUT liquid can be considered to be semi-infinite. In other words, for this level of isopropanol content (5%), Equation (14) is valid, provided the sensor is not equipped with a dry film. However, the presence of the dry film limits the validity of Equation (14), providing only approximate values. 

By considering values of the isopropanol content above 10%, Equation (20) provides good results concerning the dielectric constant of the LUT liquid, provided a dry film is not used. The reason for this is that this expression is valid, regardless of the sensor imbalance. However, the determination of the loss tangent of the LUT is not as accurate, compared to the 5% case, since the imbalance cannot be considered small. In addition, the discrepancies further increase by considering the presence of the dry film. Therefore, the determination of material (liquid) parameters through Equation (14) is limited to small perturbations and REF and LUT liquids in direct contact with the sensitive element (the dumbbell DGS). Although we have added a dry film in order to avoid absorption (as justified before), there are substrates that prevent absorption. In those cases, Equation (14) can be used to predict liquid parameters, provided that the REF and LUT liquids are not very different. Nevertheless, the determination of *ε*_LUT_ through Equation (20) is first required. Then, this parameter is introduced in Equation (14), and from this expression, and the measured cross-mode transmission coefficient, the loss tangent of the LUT can be obtained.

## 5. Conclusions

In conclusion, a real-time differential microwave sensor based on a pair of transmission lines loaded with dumbbell-shaped DGS resonators has been presented, analyzed, and validated. The sensing method is based on symmetry disruption, produced by the presence of two different materials in the sensing regions, the capacitive slots of the dumbbell DGSs. This work has focused on the characterization of liquid solutions, where the reference (REF) material has been deionized (DI) water, and the liquid under test (LUT) has included different solutions of either NaCl or isopropanol (the solutes) in DI water (the solvent). For that purpose, the sensor has been equipped with a pair of fluidic channels: one for the REF liquid and the other one for the LUT. The sensor has been optimized in order to detect tiny differences between the REF liquid and the LUT, measured through the cross-mode transmission coefficient, the output variable. For sensitivity optimization, the circuit model and an accurate analysis, from which we have derived the dependence of the output variable with the material parameters, have been the key aspects, and the main relevant contribution of this paper. It has been found that the sensitivity is optimized by designing a dumbbell DGS with narrow and long slots. The sensor has been used for the measurement of NaCl concentrations in DI water, where a resolution as good as 0.25 g/L, with a maximum sensitivity of 10.08 (dB·L/g), has been achieved. Then, the functionality of the sensor for the measurement of the complex dielectric constant of liquids (particularly solutions of isopropanol in DI water), has been demonstrated. The sensor can be used for the characterization of many other types of liquids and solutions, and it is especially useful for monitoring real-time changes in an LUT compared to an REF liquid. 

## Figures and Tables

**Table 1:** Comparison of various microwave fluidic sensors for solute concentration measurement in liquid solutions.

Reference	Max. *Sensitivity* (dB·L/g)	*Resolution* (g/L)	*Dynamic Range* (g/L)
[25]	4.3	0.25	80
[26]	12.27	0.25	60
[23]	1.609	0.5	100
[40]	0.005	2	10
[44]	0.003	1	300
[45]	1.75	1.5	5.5
[46]	0.017	10	150
[47]	0.003	5	300
[37]	6.54	0.125	60
[41]	0.822	0.5	40
[42]	7	0.0001	0.01
[43]	65	0.001	0.1
**[This work]**	**10.08**	**0.25**	**60**

**Table 2:** Comparison of various microwave fluidic sensors for dielectric characterization.

Reference	*f*_0_ (GHz)	$Diff.\;S_{av,f_{max}}/“SE”\;S_{av,f_{0}}$	*F_v_* (%)
[48]	20	-/2.98	5
[28]	2	-/2.38	10
[33]	1.9	-/0.81	10
[34]	3.5	-/2.61	10
[22]	0.87	-/0.91	10
[49]	2.5	-/3.2	0.005
[25]	0.9	1.86/2.28	5
[50]	5.5	-/0.53	0.1
[51]	5.28	-/0.193	-
[52]	2.3	-/4.08	10
[53]	2.29	-/3.26	
[54]	0.077	-/1.08	0.5
[55]	5.2	-/1.12	20
**This work**	**1.05**	**1.02/5.98**	**5**
